# Systemic calcinosis in a Quarter Horse gelding homozygous for a myosin heavy chain 1 mutation

**DOI:** 10.1111/jvim.16481

**Published:** 2022-07-08

**Authors:** Beatrice T. Sponseller, David M. Wong, Rebecca Ruby, Wendy A. Ware, Scott Wilson, Joseph S. Haynes

**Affiliations:** ^1^ Department of Veterinary Clinical Sciences College of Veterinary Medicine, Iowa State University Ames Iowa USA; ^2^ Veterinary Diagnostic Laboratory College of Agriculture, Food and Environment, University of Kentucky Lexington Kentucky USA; ^3^ Mid‐Atlantic Equine Dentistry Myrtle Beach South Carolina USA; ^4^ Department of Veterinary Pathology College of Veterinary Medicine, Iowa State University Ames Iowa USA

**Keywords:** calcium‐phosphorus product, immune mediated myositis, polydipsia, polyuria, sodium thiosulfate

## Abstract

**Case Description:**

A 9‐year‐old Quarter Horse gelding was presented for lethargy, decreased appetite, polyuria and polydipsia (PU/PD), and severe muscle wasting suggestive of immune‐mediated myositis.

**Clinical Findings:**

The horse displayed lethargy, fever, tachyarrhythmia, inappetence, PU/PD, and severe epaxial and gluteal muscle wasting. Clinicopathologic findings were consistent with previously reported cases of systemic calcinosis in horses, including increased muscle enzyme activity, hyperphosphatemia, increased calcium‐phosphorus product, hypoproteinemia, and an inflammatory leukogram. A diagnosis of systemic calcinosis was established by histopathologic evaluation of biopsy specimens from skeletal muscle, lung, and kidney.

**Treatment and Outcome:**

Symptomatic treatment was complemented by IV treatment with sodium thiosulfate to reverse calcium‐phosphate precipitation in soft tissue and PO aluminum hydroxide to decrease intestinal phosphorus absorption and serum phosphorus concentration.

**Clinical Relevance:**

This is the first report in the veterinary literature of an antemortem diagnosis of systemic calcinosis in the horse that was successfully treated and had favorable long‐term outcome.

**Abbreviations**
BSA: body surface area
FE: fractional excretion
IMM: immune‐mediated myositis
*MYH1*
: myosin heavy chain 1
PTH: parathyroid hormone
PU/PD: polyuria/polydipsia
RANK‐L: receptor activator of nuclear factor‐kappa B ligand
RI: reference interval
SG: specific gravity

## 1 INTRODUCTION

Systemic calcinosis in horses is a rare syndrome characterized by heterotopic calcification of soft tissues including skeletal muscle, heart, kidneys, lungs, and occasionally blood vessels.^1^, ^2^ To date, all affected horses described in the literature have been young (≤9 years) Quarter Horses or related breeds and the outcome has been universally fatal.^1^, ^2^ Initial clinical signs resemble immune‐mediated myositis (IMM) with rapid, severe atrophy of epaxial and gluteal muscles and generalized malaise.^1^, ^3^ Progressive clinical signs suggestive of multiple organ involvement ensue and may include tachypnea, dyspnea, tachycardia, ventral edema, progressive weakness, stiffness, and recumbency.^1^, ^4^ Clinical signs have been attributed to tissue calcification and concomitant diseases such as pleuropneumonia, tracheobronchitis, inflammatory airway disease, colitis, or renal disease.^1^, ^4^ Leukocytosis, neutrophilia, and hyperfibrinogenemia are common hematologic abnormalities.^1^, ^4^ Increased muscle enzyme activity, hyperphosphatemia, and calcium‐phosphorus product >65 are consistent clinicopathologic findings and the high calcium‐phosphorus product is thought to be the cause of ectopic calcification in affected horses.^1^, ^4^

Inflammation may be a key factor in the development of systemic calcinosis through synergistic effects of cytokines on receptor activator of nuclear factor kappa B ligand (RANK‐L)‐mediated osteoclast formation and bone resorption.^4^ The osteoclastogenic effects of inflammatory cytokines are well documented in a variety of inflammatory conditions in humans.^5^, ^6^ Hyperphosphatemia in systemic calcinosis may be caused by concurrent diseases that incite an inflammatory response that triggers inflammatory osteolysis. Other proposed causes of hyperphosphatemia and increased calcium‐phosphorus product include severe rhabdomyolysis, vitamin D toxicity, and hyperparathyroidism.^4^, ^7^

## 2 CASE REPORT

A 9‐year‐old Quarter Horse gelding was presented to Iowa State University's Lloyd Veterinary Medical Center with a 2‐week history of lethargy and decreased appetite, a 5‐day history of polyuria and polydipsia (PU/PD) and a 2‐day history of acute, severe muscle wasting. The horse had been kept individually on pasture, had no contact with other horses, and had no prior health concerns. Annual vaccinations for Eastern and Western equine encephalitis, West Nile virus, and tetanus had been given 10 months before presentation. One dose of ivermectin (200 μg/kg PO) was administered at the time of onset of lethargy.

On admission, the horse weighed 491 kg, had a body condition score of 2‐3/9, and severe epaxial and gluteal muscle atrophy suggesting IMM. Lethargy, fever (39.5°C), tachypnea (44 breaths/min), and tachyarrhythmia (50‐100 beats/min) were present. The hair coat was dull and pitting edema of the ventral thorax was evident. Other abnormalities included mild dehydration and PU/PD with hyposthenuria (specific gravity [SG] 1.003), aciduria (pH 6.5) and microhematuria.

A CBC disclosed leukocytosis (15 460/μL; reference interval [RI], 5000‐11 000/μL), mature neutrophilia (13 450/μL; RI, 2100‐6700/μL), and hyperfibrinogenemia (700 mg/dL; RI, 100‐400 mg/dL). Serum biochemistry indicated hyponatremia (128 mEq/L; RI, 137‐145 mEq/L), hypochloremia (94 mEq/L; RI, 102‐114 mEq/L), hypomagnesemia (1.27 mg/dL; RI, 1.43‐2.68 mg/dL), hypoglycemia (75 mg/dL; RI, 80‐113 mg/dL), hypoproteinemia (5.2 g/dL; RI, 5.8‐8.0 g/dL), hypoalbuminemia (1.8 g/dL; RI, 3.3‐4.6 g/dL), and mild hyperphosphatemia (5.1 mg/dL; RI, 2.6‐5.0 mg/dL). Creatine kinase activity (845 IU/L; RI, 74‐426 IU/L), aspartate aminotransferase activity (1406 IU/L; RI, 100‐465 IU/L), and BUN concentration (22 mg/dL; RI, 14‐21 mg/dL) were mildly increased. Serum creatinine concentration (1.5 mg/dL; RI, 1‐2.1 mg/dL) was within normal limits. Initial differential diagnoses included an infectious process with secondary IMM and myocarditis, ionophore toxicity, early renal failure, and diabetes insipidus.

**FIGURE 1:**
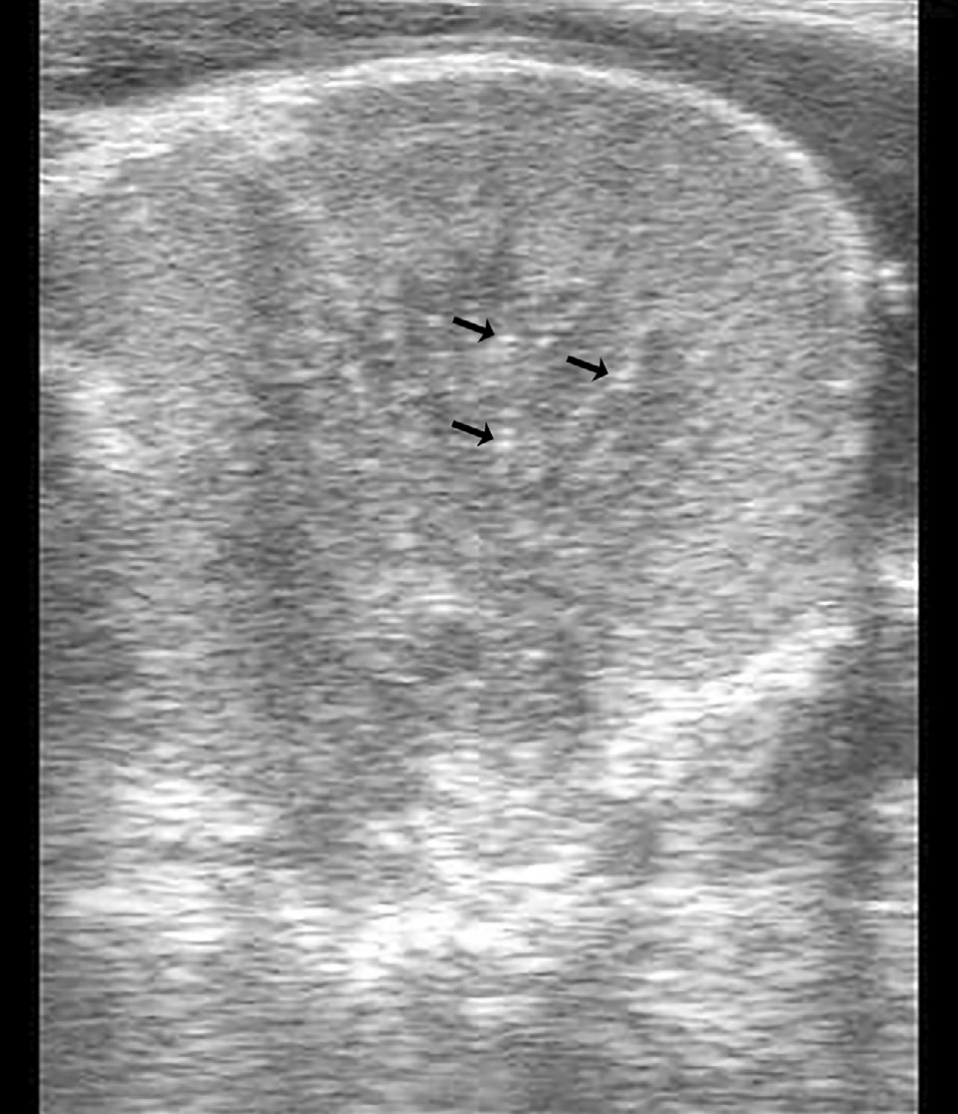
Transrectal sonogram of the left kidney showing overall increased echogenicity and fine hyperechoic speckling in the renal medulla suggestive of calcification. Image obtained with a transrectal linear 7.5 MHz probe at a maximal depth of 8 cm

**FIGURE 2:**
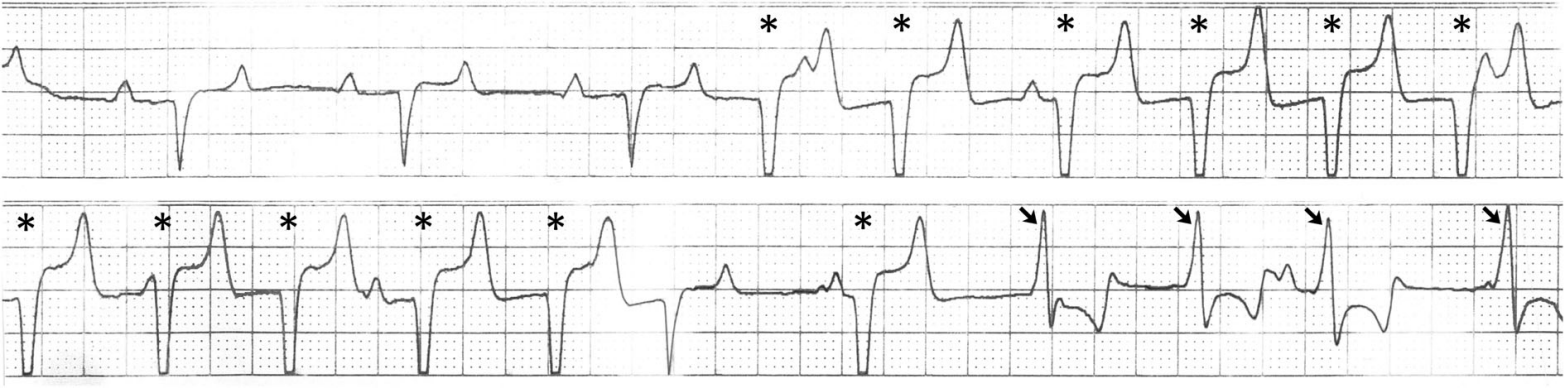
Base‐apex lead ECG tracing showing multiform ventricular tachyarrhythmia. Three initial sinus complexes are followed by a ventricular tachycardia with wide negative QRS complexes (*). A sinus complex then occurs (middle of bottom strip; P wave buried in the preceding T wave), followed by another ventricular premature (complex) and a run of positive QRS ventricular complexes (arrows). Nonconducted P waves can be seen intermittently between ectopic complexes. Paper speed 25 mm/s

Thoracic and abdominal ultrasonography were within normal limits except for increased echogenicity of both kidneys. Transrectal ultrasound evaluation of the left kidney showed hyperechogenicity of the capsule, cortex, and medulla with hyperechoic stippling along echogenic strands in the medulla (Figure 1). Thoracic radiographs disclosed a mild diffuse bronchointerstitial pattern and echocardiography indicated no structural cardiac abnormalities and normal left ventricular function. An ECG indicated sinus tachycardia with paroxysmal multiform ventricular tachyarrhythmia (Figure 2). Treatment with gentamicin sulfate (Vetone, Las Vegas, Nevada; 6.6 mg/kg IV q24h), procaine penicillin G (Vetri‐Pen G, Vetone; 22 000 IU/kg IM q12h), and flunixin meglumine (Banamine, Merck, Madison, New Jersey; 1 mg/kg IV q12h) was initiated.

On day 2 of hospitalization, pyrexia had resolved but tachypnea and tachyarrhythmia persisted. Arterial blood gas analysis identified hypoxemia (PaO_2_,70.1 mm Hg; RI, 85‐95 mm Hg) and serum cardiac troponin I concentration was increased (2.65 ng/mL; RI, <0.15 ng/mL)^8^ indicating myocardial insult. Polyuria and polydipsia continued with water consumption of 89 L/day (181 mL/kg/day; RI, 54‐83 mL/kg/day).^9^ The horse was deemed too unstable to perform a water deprivation test. Measurement of serum antidiuretic hormone (ADH) concentration was considered to evaluate for diabetes insipidus, but was not readily available. Serum biochemistry results were similar to those at presentation, with worsening hyperphosphatemia (6.1 mg/dL) and resulting increased calcium‐phosphorus product (75.6 mg^2^/dL^2^; RI, ≤65 mg^2^/dL^2^).^3^ Serum calcium concentration had increased from 10.6 to 12.4 mg/dL and, although within reference range, was considered high given the presence of severe hyopalbuminemia. Systemic calcinosis, vitamin D toxicity, and hyperparathyroidism were added to the list of differential diagnoses. Serum 25‐hydroxyvitamin D concentration was low (8 nmol/L; RI, 13‐40 nmol/L) ruling out vitamin D toxicity. Ionized calcium (1.85 mmol/L; RI, 1.58‐1.90 mmol/L) and parathyroid hormone (PTH; 6.6 PMol/L; RI, 0.6‐11 PMol/L) were within normal limits, ruling out hyperparathyroidism.

**FIGURE 3:**
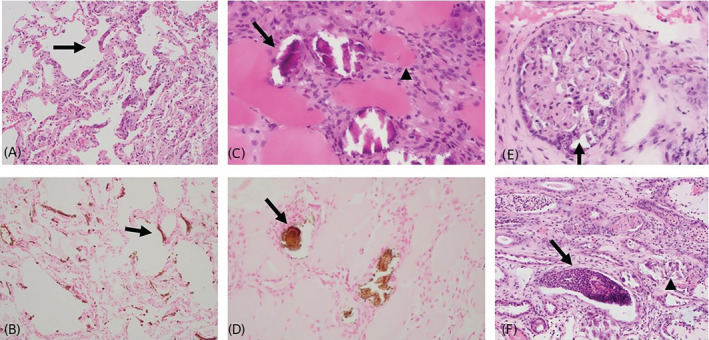
A, Lung tissue showing thickening of the alveolar wall with basophilic mineral and mononuclear cells (arrow). Hematoxylin and eosin stain, ×200 magnification. B, Areas of calcification are highlighted in the alveolar walls (arrow). Von Kossa stain, ×200 magnification. C, Biopsy specimen from the gluteus medius in a 9‐year‐old Quarter Horse. Multiple myocytes are calcified (arrow). Other myocytes are shrunken or necrotic (arrowhead). A cellular population of fibroblasts and low numbers of macrophages are present. Hematoxylin and eosin stain, ×400 magnification. D, Areas of calcification are highlighted in calcified myocytes (arrow). Von Kossa stain, ×400 magnification. E, Mineralization of the glomerular tuft and basement membrane characterized by multifocal basophilic stippling (arrow). Hematoxylin and eosin stain, ×400 magnification. F, Renal tubules contain neutrophilic and cellular debris (arrow). Adjacent tubules contain variable amounts of basophilic, crystalline material (arrow head). Hematoxylin and eosin stain, ×200 magnification

Persistently increased CK activity prompted the addition of dexamethasone (Vetone; 0.04 mg/kg IV q24h) to treat suspected IMM. Because of further increased CK activity with a peak of 1515 IU/L on day 5, the dosage was increased to 0.08 mg/kg IV q24h. Serum vitamin E and selenium concentrations were within normal limits, ruling out nutritional myodegeneration. Because systemic calcification was suspected, biopsy samples were collected from the gluteus medius muscle and lung on day 2, and liver and kidney on day 3 of hospitalization. Histopathologic evaluation identified a degenerative and mineralizing myopathy, diffuse pulmonary mineralization, and subacute to chronic purulent tubulointerstitial nephritis with focal necrosis and mineralization. A core liver specimen had no microscopic lesions. The pulmonary calcification appeared to be purely metastatic with no evidence of another cause of tissue damage or inflammation. Bronchi were not present within the biopsy sample but diffuse mineralization of the alveolar wall was observed (Figure 3A,B). Skeletal muscle displayed dystrophic calcification of myofibers, necrosis, fibrosis, and variable regeneration of myocytes consistent with previous reports of systemic calcinosis^1^, ^3^ (Figure 3C,D). Occasional clusters of lymphocytes were observed within areas of myocyte degeneration, suggestive of IMM.^3^, ^4^ Metastatic mineralization of arteriolar tunica media elastin fibers was present within sections of muscle whereas other sections had no pathologic findings. Mineralization within the renal biopsy specimen was observed within the glomerular tuft and basement membrane as well as necrotic tissue. Histopathologic lesions in the renal biopsy specimen were the only site of clinically relevant, ongoing neutrophilic inflammation, characterized by severe tubulointerstitial nephritis, acute focal necrosis, and dystrophic mineralization (Figure 3E,F).

Given the multifocal calcification and further increase in calcium‐phosphorus product (78.7 mg^2^/dL^2^), sodium thiosulfate (American Regent, Shirley, New York; 50 mg/kg, diluted to 10% in 5% dextrose, slowly IV q48h) was added to the treatment regimen on day 4 of hospitalization because of its proposed anti‐calcification properties in human patients with extraosseus calcification. Additionally, aluminum hydroxide (Rugby Laboratories, Livonia, Michigan; 30 mg/kg [15 g] PO q24h) was administered beginning on day 5 to decrease dietary phosphorus absorption and the calcium‐phosphorus product. Hematologic parameters had improved with persistent mild mature neutrophilia (7200/μL) and hyperfibrinogenemia (500 mg/dL). At that time, antimicrobial and anti‐inflammatory medications were changed to sulfamethoxazole‐trimethoprim (Qualitest Pharmaceuticals, Huntsville, Alabama; 30 mg/kg PO q12h) and pentoxifylline (Trental, Sanofi‐Aventis, Bridgewater, New Jersey; 7.5 mg/kg PO q12h), respectively.

Tachypnea resolved within 3 and tachyarrhythmia within 6 days after admission, and repeat blood gas analysis was within normal limits. Sequential serum biochemistry analyses showed normalization of serum phosphorus concentration on day 8 (4.2 mg/dL) and hypophosphatemia (2.0 mg/dL) on day 10, with normal calcium‐phosphorus product of 53 and 25 mg^2^/dL^2^, respectively, prompting discontinuation of aluminum hydroxide administration. Serum sodium and total protein concentrations had normalized and hypoalbuminemia had improved (2.4 g/dL), whereas serum chloride concentration and CK and AST activities approached normal limits.

Polyuria and polydipsia were persistent features with initial water consumption of approximately 181 mL/kg/day that decreased to 119 mL/kg/day over 10 days of hospitalization. Serum creatinine concentration remained within normal limits whereas BUN peaked on day 3 (26 mg/dL) before returning to normal. Fractional excretion (FE) of sodium in urine was measured on day 4 to assess renal tubular function because it may be a more sensitive diagnostic tool to document early renal dysfunction in the absence of azotemia.^10^ Results were within normal limits (0.69%; RI, <1**%**).^10^ Urine SG increased to 1.008 (isosthenuria) and urine pH (7.5) had normalized by day 5 of hospitalization with only a trace of blood registering on a urine dipstick. Urinalysis on day 10 was unchanged. A final dose of sodium thiosulfate was given that day before discharging the horse with instructions to continue treatment with PO pentoxifylline for 3 days and sulfamethoxazole‐trimethoprim and dexamethasone (0.08 mg/kg and decreasing daily by 0.004 mg/kg) for 3 weeks.

**FIGURE 4:**
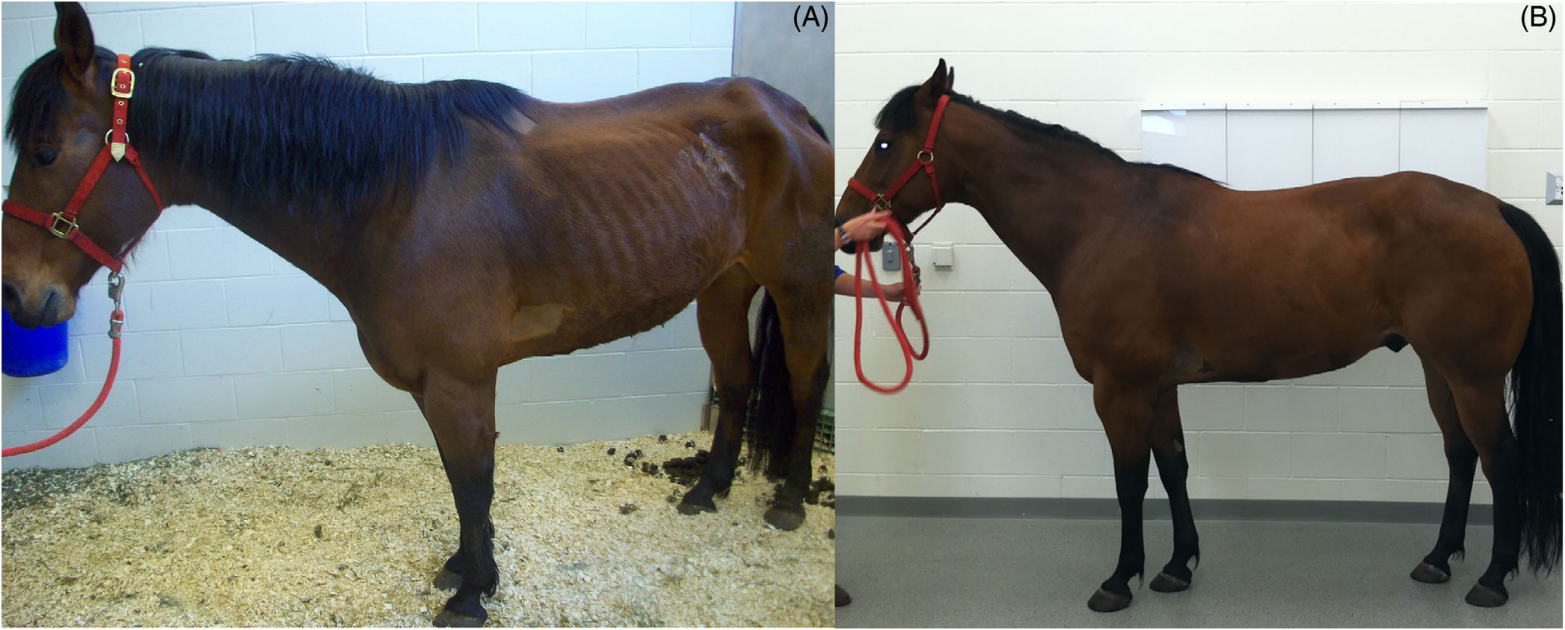
A, Patient on the day of presentation. Notice the marked epaxial and gluteal muscle atrophy. B, Patient 11 weeks after initial presentation. Notice the marked improvement in overall body condition, especially the epaxial and gluteal muscle mass

Five days after discharge, the horse was readmitted and hospitalized for 10 days because of decreased appetite, intermittent bruxism, and mild signs of colic that responded to treatment with omeprazole (GastroGard, Merial, Duluth, Georgia; 4 mg/kg PO q24h for 14 days). Additional doses of 10% sodium thiosulfate were given 1 day and 4 days after readmission. Polyuria, polydipsia (102‐118 mL/kg/day), isosthenuria (SG, 1.013), and mild microhematuria persisted, whereas FE of sodium (0.147%) remained normal. Activities of CK and AST had normalized at readmission and 1 week thereafter, respectively. Five weeks after initial presentation, urinalysis was within normal limits with hypersthenuria (SG, 1.021), indicating that the kidneys were actively concentrating urine, and no evidence of hematuria. Water intake had normalized at approximately 80 mL/kg/day. Anemia (PCV, 24.2%; RBC, 5.23 × 10^6^/μL) was the only abnormality on a CBC. The horse had a good appetite and attitude and was active during limited pasture turnout. Eleven weeks after initial presentation epaxial and gluteal muscle atrophy had markedly improved (Figure 4). Echocardiographic and renal ultrasonographic reevaluation disclosed no abnormalities. Hypoalbuminemia (2.8 g/dL) and anemia (PCV, 30.6%; RBC, 6.58 × 10^6^/μL) had improved whereas urinalysis remained within normal limits with a SG of 1.027. Thirty weeks after the initial presentation, epaxial and gluteal muscle atrophy had resolved and biopsy of the gluteus medius muscle identified no histopathologic abnormalities. The gelding continued to do well at home and a CBC and serum biochemistry profile 3 years after the initial presentation were within normal limits. Genetic testing 8 years after initial presentation identified homozygosity for a myosin heavy chain 1 (*MYH1*) mutation indicating increased susceptibility to IMM.^11^

## 3 DISCUSSION

Systemic calcinosis in horses can be difficult to diagnose because of its variable clinical presentation and multiorgan involvement and hitherto has been an exclusively postmortem diagnosis.^1^, ^2^ This syndrome is thought to have an immune‐mediated component with clinical signs of IMM and a myriad of concomitant or possibly triggering disease processes.^4^ Our case had clinical signs of IMM and serious multiple organ involvement, including the respiratory, cardiovascular, and urinary systems. A diagnosis of systemic calcinosis was achieved by tissue biopsy and histopathologic documentation of severe calcification in the lung, kidney, and skeletal muscle. Skeletal muscle also showed occasional lymphocytic infiltrates suggestive of IMM.^3^, ^4^ Metastatic mineralization of arteriolar tunica media elastin fibers was present within sections of muscle and may have caused ischemic necrosis and dystrophic calcification. Despite these abnormal findings, CK activity was only mildly and transiently increased in our patient. It is possible that muscle damage was restricted to a smaller set of muscle groups or focal rather than diffuse within affected muscle. Furthermore, histopathologic changes in the gluteus medius were chronic and not highly inflammatory. In fact, the kidney was the only site of clinically relevant inflammation, characterized by purulent tubulointerstitial nephritis, acute focal necrosis, and mineralization. Medullary calcification can cause PU/PD^12^ and, in conjunction with distal tubular damage and resulting impaired urine concentrating ability, was likely the cause of severe PU/PD in our patient.

The heart is another common site of calcification in systemic calcinosis in horses,^1^ which may have accounted for the tachyarrhythmia and increased troponin I concentration in our patient. Immune‐mediated myocarditis also was considered given prior documentation of increased troponin I concentration and tachycardia in 2 horses with IMM.^13^ Predilection sites for metastatic calcification in human patients are similar,^14^, ^15^ although skeletal muscle calcification is rare and has only been reported in the context of calciphylaxis.^16^ Calciphylaxis is a specific form of metastatic calcification characterized by mural calcification and thrombosis of small arteries with subsequent tissue necrosis.^15^ Systemic calciphylaxis with skeletal muscle calcification and degenerative myopathy also has been reported in a young cat with idiopathic hypercalcemia.^17^ An increased calcium‐phosphorus product has been invoked to explain the pathogenesis of calciphylaxis and systemic calcinosis. Furthermore, chronic renal failure, increased PTH, and hyperphosphatemia are known risk factors for the development of metastatic calcification in humans.^14^, ^15^ No primary cause of hyperphosphatemia and increased calcium‐phosphorus product was identified in our patient. Acute renal failure was considered given the severe histopathologic lesions and PU/PD. However, renal function test results remained within normal limits. Normal PTH activity ruled out secondary hyperparathyroidism and the increase in muscle enzyme activities was deemed too mild for rhabdomyolysis‐induced hyperphosphatemia. Vitamin D intoxication consistently causes hyperphosphatemia, PU/PD, and soft tissue calcification,^7^ but vitamin D concentration was low in our case. Given the fever, neutrophilia, and hyperfibrinogenemia on presentation, inflammatory osteolysis was considered a plausible cause of hyperphosphatemia, with purulent tubulointerstitial nephritis being the most likely source of inflammation. Immune‐mediated myositis also has been associated with fever and an inflammatory leukogram^13^ and may have served as an additional source of inflammation. In addition to upregulating osteoclastic activity, inflammation may promote soft tissue calcification by downregulating fetuin‐A, a hepatic glycoprotein and potent inhibitor of ectopic calcification.^18^

Early diagnosis in our case allowed targeted treatment aiming at halting and reversing soft tissue calcification by PO administration of phosphate binders and IV administration of sodium thiosulfate. Sodium thiosulfate has been used in the treatment of pathologic calcification in humans, including tumoral calcinosis, characterized by deposition of calcium phosphate microcrystals in periarticular tissues,^19^ and calciphylaxis.^20^, ^21^ The empirical dose is 12.5‐25 g given IV 3 times per week^19^, ^22^ or 12‐25 g/1.72m^2^ per dose.^23^, ^24^ Given a body surface area (BSA) estimate of 5 m^2^ for a 491 kg horse (BSA = 1.09 + 0.008 × body weight [kg]),^25^ the dose range for our patient was calculated to be 35‐73 g (71‐149 mg/kg). However, given published dosages of 30‐40 mg/kg sodium thiosulfate for cyanide poisoning in horses,^26^ we chose a dose of 25 g (50 mg/kg), equal to the maximum dose for adult humans. Sodium thiosulfate is fairly nontoxic and safe if given slowly IV, although in humans, adverse effects of nausea, vomiting and metabolic acidosis have been reported.^27^ After distribution to the extracellular space, the drug is rapidly excreted in urine.^26^ The reduction or resolution of ectopic calcification has been attributed to potent calcium chelating properties of sodium thiosulfate through formation of highly soluble calcium thiosulfate,^27^ although this mechanism recently has been disputed, and the exact mechanism of action remains unknown.^19^, ^28^ Vasodilatory, anti‐inflammatory and antioxidant effects may contribute to the therapeutic effects of sodium thiosulfate.^28^, ^29^ It appears that sodium thiosulfate contributed to the resolution of systemic calcinosis and a positive outcome in our case. Furthermore, the normalization of serum phosphorus concentration and calcium‐phosphorus product within 3 days of PO aluminum hydroxide administration suggests that phosphate binders also are useful in the treatment of systemic calcinosis. Aluminum hydroxide is a heavy metal salt that decreases intestinal phosphate absorption by forming insoluble complexes with dietary phosphate that are excreted through the intestinal tract.^30^ Decreasing serum phosphorus concentration by use of PO phosphate binders is a mainstay in the treatment and prevention of ectopic calcification in humans.^31^ Off‐label use of aluminum hydroxide in dogs and cats at a dosage of 30‐100 mg/kg/day is common and adverse effects are rarely reported, although aluminum toxicity causing severe neuromuscular deficits was documented in 2 dogs with chronic kidney disease treated with dosages >100 mg/kg/day.^32^ Given the lack of published data in horses and lower bioavailability of plant‐derived phosphorus (phytate) in a vegetarian diet compared to the organic phosphate in animal protein,^33^ a low dosage of aluminum hydroxide (30 mg/kg/day) was chosen for our patient.

The use of glucocorticoids in the treatment of systemic calcinosis is controversial given their pro‐osteoclastic effects. Glucocorticoids increase expression of RANK‐L and prolong osteoclast life span resulting in enhanced bone resorption and possibly worsening of hyperphosphatemia.^34^, ^35^ However, their potent anti‐inflammatory action may prove beneficial if indeed a systemic inflammatory process is triggering systemic calcinosis. The suspicion of IMM contributing to the pathogenesis of systemic calcinosis prompted immunosuppressive treatment with dexamethasone in our patient. Recently, a missense mutation in the *MYH1* gene, coding for the fast skeletal myosin heavy chain type 2X that predisposes to IMM, has been identified in Quarter Horses and related breeds.^11^ Affected horses are at risk of developing IMM after respiratory disease, vaccination, or other often unidentified triggers.^4^ No triggering event was identified in our patient. Compared to *MYH1* heterozygotes, homozygotes appear to be more commonly affected with IMM and display hypercontractility of type 2X fibers, which may trigger an auto‐immune response via cellular damage and exposure of affected myosin.^36^ To date, it remains unclear why some horses with IMM develop systemic calcinosis. All horses with IMM and systemic calcinosis had at least 1 other systemic inflammatory condition.^1^, ^4^ The coexistence of more than 1 condition could lead to an inflammatory response potent enough to induce inflammatory osteolysis, setting off the cascade of hyperphosphatemia and increased calcium‐phosphorus product that precedes soft tissue calcification.

## CONFLICT OF INTEREST DECLARATION

Authors declare no conflict of interest.

## OFF‐LABEL ANTIMICROBIAL DECLARATION

Gentamicin sulfate, aluminum hydroxide and sodium thiosulfate was used off‐label.

## INSTITUTIONAL ANIMAL CARE AND USE COMMITTEE (IACUC) OR OTHER APPROVAL DECLARATION

Authors declare no IACUC or other approval was needed.

## HUMAN ETHICS APPROVAL DECLARATION

Authors declare human ethics approval was not needed for this study.
